# Efficacy of preoperative biliary drainage in malignant obstructive jaundice: a meta-analysis and systematic review

**DOI:** 10.1186/s12957-016-0933-2

**Published:** 2016-07-11

**Authors:** Harsha Moole, Matthew Bechtold, Srinivas R. Puli

**Affiliations:** Division of General Internal Medicine, University of Illinois College of Medicine at Peoria, Peoria, IL USA; Department of Gastroenterology and Hepatology, University of Missouri, Columbia, MO USA; Division of Gastroenterology and Hepatology, University of Illinois College of Medicine at Peoria, Peoria, IL USA; Department of Medicine, University of Illinois College of Medicine Peoria, 530 NE Glen Oak Ave, Peoria, IL 61637 USA

**Keywords:** Preoperative biliary drainage, Malignant obstructive jaundice, Pancreatic head cancer, Peri-ampullary malignancy, Cholangiocarcinoma, Pancreaticoduodenectomy, Malignant biliary stricture, Meta-analysis, Systematic review

## Abstract

**Background:**

In patients requiring surgical resection for malignant biliary jaundice, it is unclear if preoperative biliary drainage (PBD) would improve mortality and morbidity by restoration of biliary flow prior to operation. This is a meta-analysis to pool the evidence and assess the utility of PBD in patients with malignant obstructive jaundice. The primary outcome is comparing mortality outcomes in patients with malignant obstructive jaundice undergoing direct surgery (DS) versus PBD. The secondary outcomes include major adverse events and length of hospital stay in both the groups.

**Methods:**

Studies using PBD in patients with malignant obstructive jaundice were included in this study. For the data collection and extraction, articles were searched in MEDLINE, PubMed, Embase, Cochrane Central Register of Controlled Trials & Database of Systematic Reviews, etc. Pooled proportions were calculated using both Mantel-Haenszel method (fixed effects model) and DerSimonian-Laird method (random effects model).

**Results:**

Initial search identified 2230 reference articles, of which 204 were selected and reviewed. Twenty-six studies (*N* = 3532) for PBD in malignant obstructive jaundice which met the inclusion criteria were included in this analysis. The odds ratio for mortality in PBD group versus DS group was 0.96 (95 % CI = 0.71 to 1.29). Pooled number of major adverse effects was lower in the PBD group at 10.40 (95 % CI = 9.96 to 10.83) compared to 15.56 (95 % CI = 15.06 to 16.05) in the DS group. Subgroup analysis comparing internal PBD to DS group showed lower odds for major adverse events (odds ratio, 0.48 with 95 % CI = 0.32 to 0.74).

**Conclusions:**

In patients with malignant biliary jaundice requiring surgery, PBD group had significantly less major adverse effects than DS group. Length of hospital stay and mortality rate were comparable in both the groups.

## Background

Malignancies obstructing the biliary tract or ampulla of Vater can cause obstructive jaundice. Close to 20 % of these malignancies are resectable at the time of presentation [1–3]. These malignancies include cancers of the biliary tract, cancers of the head and neck of the pancreas, cancers of the second part of duodenum, and cancer of the ampulla of Vater. Biliary obstruction alters the normal physiology and affects multiple organ systems that include but are not limited to cardiac, renal, hematologic, and hepatic dysfunction [4–7]. Hyperbilirubinemia is a potential risk factor that might be associated with poor surgical outcomes [8–10].

Evidence suggests that biliary drainage may improve immune function and nutritional status and reduce the risk of infection [11–13]. Patients with malignant obstructive jaundice undergoing surgery are at increased risk of postoperative complications [14, 15]. As an attempt to reduce these complications, preoperative biliary drainage was pursued in these patients. The idea was to restore normal physiology by improving the biliary drainage. The “Whipple’s surgery” in 1935 was a two-staged surgery intended to facilitate the same, i.e., biliary drainage [16]. Numerous studies have been published since then that evaluated the efficacy of preoperative biliary drainage in these patients.

Preoperative biliary drainage (PBD) can be achieved by an internal or external approach. Internal biliary drainage is achieved by endoscopic placement of a biliary stent and endoscopic sphincterotomy. External biliary drainage is performed via a fluoro-guided percutaneous transhepatic approach. Previous studies have shown no mortality benefit from preoperative biliary drainage in these patients; however, it was associated with increased morbidity [17, 18]. As mentioned earlier, there is no evidence of benefits from the use of preoperative biliary drainage. This being said, as many as 6 out of 10 patients still end up getting some sort of biliary drainage procedure [19]. It is done as a temporary measure to relieve jaundice in patients waiting for surgery (either due to prolonged waiting period or delay due to preoperative assessment) and post-endoscopic retrograde cholangiopancreatography prophylactic biliary stent placement to prevent cholangitis.

The most recent meta-analysis by Fang et al. [17] included only randomized controlled trials (RCT). Meta-analysis done by Sewnath et al. [18] in 2002 included RCTs and retrospective cohort studies. Newer studies have been published that have not been included in the prior meta-analyses. In our meta-analysis, we sought to include all the available studies [20–45] including RCTs and retrospective cohort studies evaluating the efficacy of preoperative biliary drainage in patients with malignant obstructive jaundice. The primary outcome is to compare mortality in patients with malignant obstructive jaundice undergoing direct surgery (DS) versus preoperative biliary drainage. The secondary outcomes include major adverse events (morbidity) and length of hospital stay in both the groups.

## Methods

### Study selection criteria

Studies using preoperative biliary drainage in patients with malignant obstructive jaundice were included in this study. Biliary drainage should have been achieved via an internal approach, an external approach, or both. Studies must have compared outcomes in both the wings. Studies that looked only at one wing (non-comparison studies) were excluded from this analysis.

### Data collection and extraction

Articles were searched in MEDLINE, PubMed, Ovid journals, Embase, Cumulative Index for Nursing and Allied Health Literature, ACP Journal Club, DARE, International Pharmaceutical Abstracts, old MEDLINE, MEDLINE Non-Indexed Citations, OVID Healthstar, and Cochrane Central Register of Controlled Trials (CENTRAL). The search was performed for the years 1966 to December 2015. Abstracts were manually searched in the major gastroenterology journals for the past 3 years. Study authors for the abstracts included in this analysis were contacted when the required data for the outcome measures could not be determined from the publications. The search terms used were preoperative biliary drainage, malignant obstructive jaundice, pancreatic head cancer, peri-ampullary malignancy, cholangiocarcinoma, pancreaticoduodenectomy, malignant biliary stricture, mortality, morbidity, length of hospital stay, complications, meta-analysis, and systematic review. Two authors (HM and SP) independently searched and extracted the data into an abstraction form. Any differences were resolved by mutual agreement. The agreement between reviewers for the collected data was quantified using the Cohen’s *k* [46].

### Quality of studies

Clinical trials designed with a control and treatment arms can be assessed for quality of the study. A number of criteria have been used to assess this quality of a study (e.g., randomization, selection bias of the arms in the study, concealment of allocation, and blinding of outcome) [47, 48]. There is no consensus on how to assess studies designed without a control arm. Hence, these criteria do not apply to studies without a control arm [48].

### Statistical methods

This meta-analysis was performed by calculating pooled proportions. First the individual study proportion of mortality rates, overall major adverse effects, and length of hospital stay were transformed into a quantity using Freeman-Tukey variant of the arcsine square root transformed proportion. The pooled proportion is calculated as the back-transform of the weighted mean of the transformed proportions, using inverse arcsine variance weights for the fixed effects model and DerSimonian-Laird weights for the random effects model [49, 50]. Forest plots were drawn to show the point estimates in each study in relation to the summary pooled estimate. The width of the point estimates in the forest plots indicates the assigned weight to that study. The heterogeneity among studies was tested using Cochran’s *Q* test based upon inverse variance weights [51]. If the *p* value is >0.10, it rejects the null hypothesis that the studies are heterogeneous. The effect of publication and selection bias on the summary estimates was tested by both Harbord-Egger bias indicator [52] and Begg-Mazumdar bias indicator [53]. Also, funnel plots were constructed to evaluate potential publication bias using the standard error and diagnostic odds ratio [54, 55]. Microsoft Excel 2013 software was used to perform statistics for this meta-analysis.

## Results

**Fig. 1:**
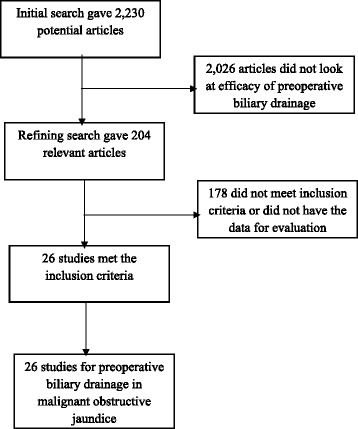
Flow chart with search results and selection criteria

Initial search identified 2230 reference articles, of which 204 articles were selected and reviewed. Data was extracted from 26 studies (*N* = 3532) that looked at preoperative biliary drainage in malignant obstructive jaundice which met the inclusion criteria. All the studies are published as full-text articles. Figure 1 shows the search results. All the pooled estimates given are estimates calculated by the fixed and random effect models.

Table 1 shows the baseline characteristics of the studies. Of the 26 studies included in this meta-analysis, eight [21, 29, 35, 36, 40, 41, 43, 44] are randomized controlled trials, one study is a prospective cohort analysis [20], one study is a single-centered observatory study [23], and the rest are retrospective studies. The pooled effects estimated by fixed and random effect models were similar. The *p* for chi-squared heterogeneity for all the pooled accuracy estimates was >0.10.

**Table 1:** Basic characteristics of the included studies

Number	Study/year	Country	Type of study	Type of drainage	Sex, M/F	Total number of patients in the study	N-PBD	N-DS
1	Morris-Stiff et al. 2011 [20]	UK	Prospective cohort analysis	Internal and External	152/128	280	118	162
2	van der Gaag et al. 2010 [21]	USA	Randomized controlled trial	Internal and External	119/83	202	106	96
3	Coates et al. 2009 [22]	USA	Retrospective cohort analysis	Internal and External	48/42	90	56	34
4	Anderson et al. 2004 [23]	Republic of South Africa	Single center observational study	Internal and External	4/2	6	6	0
5	Pisters et al. 2001 [24]	USA	Retrospective cohort analysis	Internal and External	164/136	300	207	93
6	Sewnath et al. 2001 [25]	Netherlands	Retrospective cohort analysis	Internal	148/142	290	232	58
7	Martignoni et al. 2001 [26]	Switzerland	Retrospective cohort analysis	Internal and external	140/117	257	99	158
8	Sohn et al. 2000 [27]	USA	Retrospective cohort analysis	Internal and External	297/270	567	408	159
9	Figueras et al. 2000 [28]	Spain	Retrospective cohort analysis	External	−	20	11	9
10	Wig et al. 1999 [29]	India	Randomized controlled trial	External	20/20	40	20	20
11	Povoski et al. 1999 [30]	USA	Retrospective cohort analysis	Internal and external	134/106	240	126	114
12	Hochwald et al. 1999 [31]	USA	Retrospective cohort analysis	Internal and external	40/31	71	42	29
13	Heslin et al. 1998 [32]	USA	Retrospective cohort analysis	Internal and external	41/33	74	39	35
14	Marcus et al. 1998 [33]	USA	Retrospective cohort analysis	Internal	32/20	52	22	30
15	Karsten et al. 1996 [34]	Netherlands	Retrospective cohort analysis	Internal and external	152/89	241	184	57
16	Chou et al. 1996 [35]	Taiwan	Randomized controlled trial	Internal and external	50/43	93	26	67
17	Lai et al. 1994 [36]	Hong Kong	Randomized controlled trial	Internal	59/28	87	43	44
18	Bakkevold et al. 1993 [37]	Norway	Retrospective cohort analysis	Internal and external	−	108	35	73
19	Sirinek and Levine 1989 [38]	USA	Retrospective cohort analysis	Internal and external	−	138	84	54
20	Lygidakis et al. 1987 [39]	Netherlands	Retrospective cohort analysis	Internal	21/17	38	19	19
21	Pitt et al. 1985 [40]	USA	Randomized controlled trial	Internal and external	45/30	79	37	38
22	Smith et al. 1985 [41]	Australia	Randomized controlled trial	Internal and external	20/10	30	15	15
23	Gundry et al. 1984 [42]	USA	Retrospective cohort analysis	Internal and external	−	50	25	25
24	McPherson et al. 1984 [43]	UK	Randomized controlled trial	External	−	65	34	31
25	Hatfield et al. 1982 [44]	UK	Randomized controlled trial	External	−	57	28	27
26	Denning et al. 1981 [45]	USA	Retrospective cohort analysis	Internal and external	29/28	57	25	32

*N*-*PBD* number of patients in the preoperative biliary drainage group, *N*-*DS* number of patients in direct surgery group

### Mortality and morbidity

**Fig. 2:**
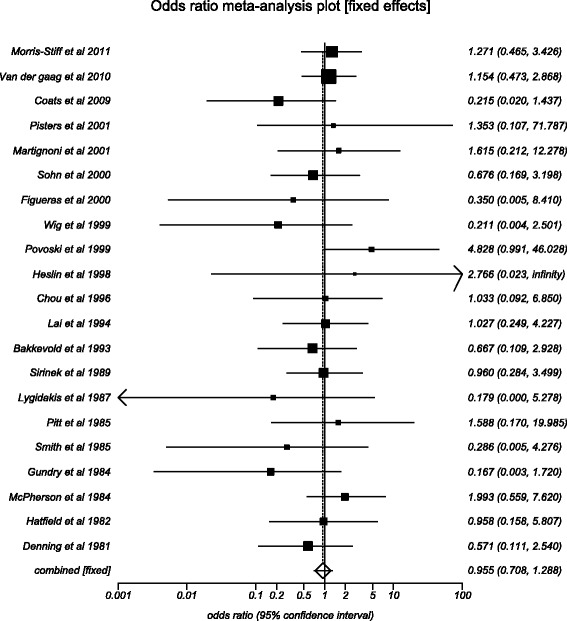
Forest plot: individual study proportions and the pooled estimate of odds ratio for mortality in the PBD group versus the DS group. (Fixed effects)

**Fig. 3:**
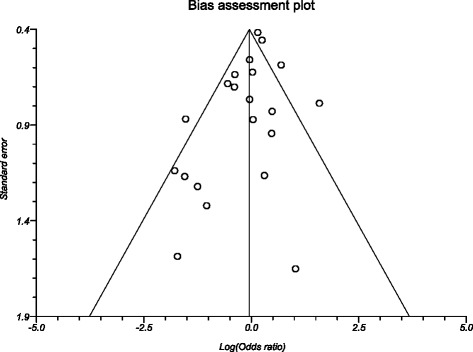
Funnel plot for publication bias assessment (odds ratio for mortality)

The odds of mortality in the preoperative biliary drainage group compared to direct surgery group was 0.96 (95 % CI = 0.71 to 1.29). Figure 2 shows the forest plot showing odds in individual studies. Publication bias calculated using Harbord-Egger bias indicator gave a value of −1.04 (95 % CI = −2.22 to 0.15, *p* = 0.08). The Begg-Mazumdar indicator gave a Kendall’s tau *b* value of −0.23 (*p* = 0.14). Figure 3 shows the funnel plot for bias. The agreement between reviewers for the collected data gave a Cohen’s *k* value of 1.0.

**Fig. 4:**
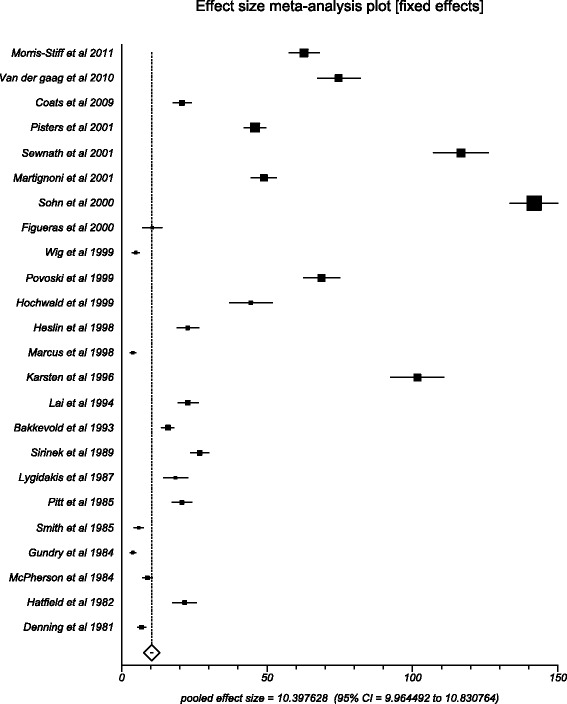
Forest plot: individual study proportions and the pooled estimate of effect size for overall adverse events in the PBD group

**Fig. 5:**
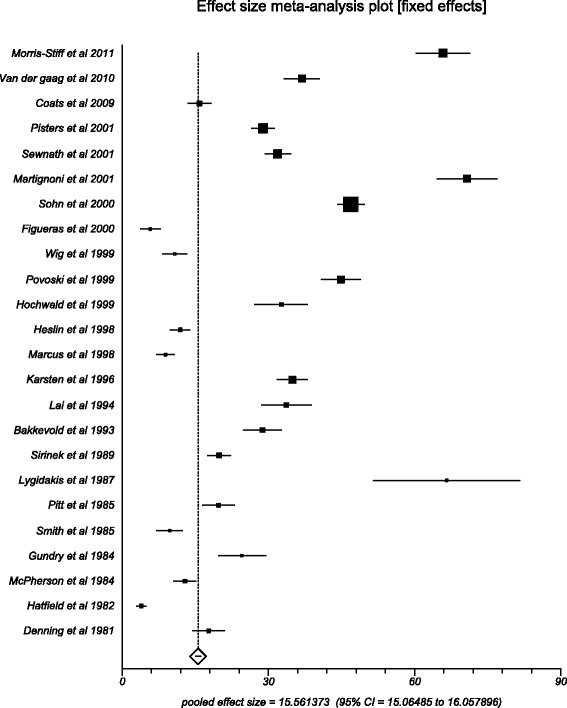
Forest plot: individual study proportions and the pooled estimate of effect size for overall adverse events in the DS group

Major adverse events include pancreatitis, cholangitis, perforation, stent occlusion, pancreaticojejunostomy leakage, gastrojejunostomy leakage, duodenojejunostomy leakage, hemorrhage after ERCP or pancreatectomy, biliary leakage, delayed gastric emptying, myocardial infarction, portal vein thrombosis, wound infection, pneumonia, need for repeated laparotomy, and intraabdominal abscess. Pooled number of major adverse events in preoperative biliary drainage group was 10.40 (95 % CI = 9.96 to 10.83) compared to 15.56 (95 % CI = 15.06 to 16.05) in the direct surgery group. The Begg-Mazumdar indicator gave a Kendall’s tau *b* value of 0.69 (*p* = <0.0001) and 0.49 (*p* = 0.0006) in the PBD and DS groups, respectively. Figures 4 and 5 show the forest plots of the major adverse event effect size in individual studies, in the PBD and DS groups, respectively.

### Length of hospital stay

Pooled fixed effect size of length of postoperative hospital stay in preoperative biliary drainage followed by surgery group was 15.20 days (95 % CI = 14.56 to 15.82) compared to 16.20 days (95 % CI = 15.30 to 17.05) in the direct surgery group. The Begg-Mazumdar indicator gave a Kendall’s tau *b* value of 0.67 (*p* = 0.013) and 0.67 (*p* = 0.013) in the PBD and DS groups, respectively. Publication bias calculated using Harbord-Egger bias indicator gave a value of 5.3 (95 % CI = 1.87 to 8.73, *p* = 0.08) and 5.5 (95 % CI = 2.03 to 8.90, *p* = 0.007) in the PBD and DS groups, respectively.

### Internal PBD versus DS subgroup analysis

**Fig. 6:**
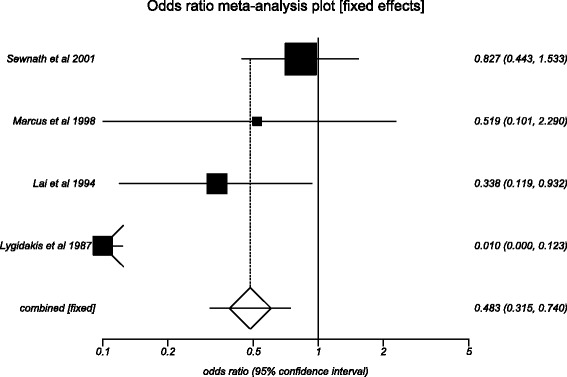
Forest plot: individual study proportions and the pooled estimate of odds ratio for major adverse events in the internal PBD group versus the DS group. (Fixed effects)

Subgroup analysis was performed on studies that compared internal PBD and DS in patients with malignant biliary strictures. Four studies [25, 33, 36, 39] were included in this analysis. The total number of patients in this subgroup (*n*) was 467, with 316 patients in the internal PBD group. The median age of patients in this subgroup was 65 years, with predominantly male population (64 %). The pooled odds ratio for major adverse effects in the internal PBD group compared to the DS group was 0.48 (95 % CI = 0.32 to 0.74). Figure 6 is a forest plot that displays the same data. Due to the limited data available, we were not able to generate any meaningful results in regard to the mortality and length of hospital stay.

### External/percutaneous transhepatic PBD versus DS subgroup analysis

**Fig. 7:**
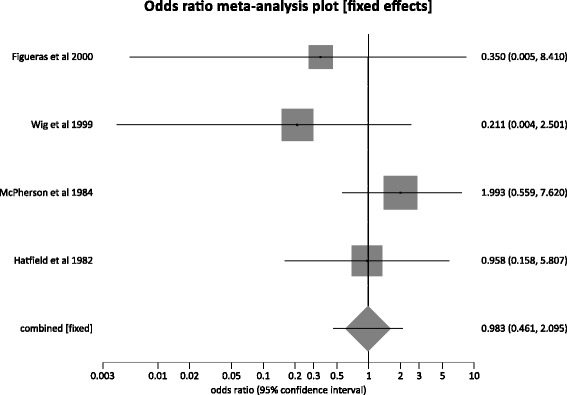
Forest plot: individual study proportions and the pooled estimate of odds ratio for mortality in the external PBD group versus the DS group. (Fixed effects)

Subgroup analysis was also performed on studies that compared percutaneous transhepatic PBD and DS in patients with malignant biliary strictures. Four studies [28, 29, 43, 44] were included in this analysis. The total number of patients in this subgroup (*n*) was 182, with 93 patients in the percutaneous transhepatic PBD group. The median age of patients in this subgroup was 62 years, with predominantly male population (61 %). The pooled odds ratio for mortality in the percutaneous transhepatic PBD group compared to the DS group was 0.98 (95 % CI = 0.46 to 2.10). *I*^2^ (inconsistency) = 21.4 % (95 % CI = 0 % to 74.2 %). Egger, bias = −2.98 (95 % CI = −5.80 to −0.15) *p* = 0.04. Figure 7 is a forest plot that displays the same data. Odds ratio for major adverse events in percutaneous transhepatic PBD group versus DS group was 1.50 (95 % CI = 0.85 to 2.65). *I*^2^ (inconsistency) = 88.5 % (95 % CI = 68.8 to 93.8 %). Egger, bias = 3.70 (95 % CI = −18.41 to 25.81) *p* = 0.54. Due to the limited data available, we were not able to generate any meaningful results in regard to the length of hospital stay and individual adverse events.

## Discussion

Based on literature review, it is unclear if PBD is beneficial to the patients with malignant biliary strictures. On the contrary, this analysis showed that the PBD group might be associated with the overall less major adverse events compared to the DS group. The internal PBD group had statistically significant reduction in the major adverse events compared to the DS group. There was no difference in mortality and length of hospital stay in both the groups

Surgical procedures to treat these malignancies are associated with high complication rates even with good surgical expertise at high volume centers. With the advancements in medical and surgical fields, the postoperative complications have relatively come down. van der Gaag et al. [21] showed that the PBD group was associated with slightly lower surgery related mortality compared to the DS group; however, there was no difference in the overall survival time.

Biliary stenting attempts at restoring the normal physiology. Sometimes even with stenting, the bilirubin level and hepatic function may not come back to normal due to the inadequate duration of stenting or due to the damage already done by the malignant obstruction. The trials had a wide range (10–32 days) of duration of PBD. The longer duration could be from the extended waiting time for surgery or due to the slow improvement in hepatic function with PBD. After a biliary stent has been placed, hepatic function takes close to 4–6 weeks to return to normal even though the bilirubin levels might fall more quickly. There are studies that showed that some of the physiological functions might not return to baseline even after 6 weeks of stenting [43, 56–59]. Due to the nature of data that was available from the trials, we were unable to perform a subgroup analysis based on the duration of PBD. Shorter duration stenting might not provide enough time to completely reverse the hepatic function. On the other hand, longer duration of stenting could be associated with stenting-related complications like stent occlusion, infection, migration, and tumor progression.

In a meta-analysis of randomized controlled trials done by Fang et al. [17], PBD group showed no mortality benefit however was associated with increased morbidity compared to direct surgery group. Another meta-analysis by Sewnath et al. [18] concluded on almost similar results. However, our meta-analysis shows that the PBD group was associated with the overall less major adverse events compared to the direct surgery group, especially in patients undergoing internal PBD. A recent published article [60] mentioned certain clinical situations where PBD could be indicated when patients had unresectable cancers or there has been a delay in surgery.

There are a few limitations to this analysis. Studies included have used plastic stents and self-expandable metal stents. Plastic stents are associated with increased number of complications and short patency. Due to the paucity of data, we were not able to compare outcomes in metallic versus plastic stents [61–67]. Different approaches of stenting have been used. Internal stenting was used in four studies [25, 33, 36, 39], and external stenting was used in four studies [28, 29, 43, 44] whereas internal and external stenting have been used in rest of the studies [20–24, 26, 27, 30–32, 34, 35, 37, 38, 40–42, 45]. Suboptimal ERCP expertise in certain studies could have affected the outcomes. Few studies included in this analysis are old, done in 1980s. The surgical procedures, stenting techniques, and stent types used in these old studies could be outdated and associated with increased complications. Different types of surgical techniques have been used in these trials. Bypass procedure has been shown to have better outcomes compared to pancreatoduodenectomy. There is no clear definition for the optimal duration of PBD prior to surgery. Some authors however support that 4 weeks is an optimal time duration for PBD. Most of the studies have unclear allocations, and reasons for drop outs from the trials were unclear. The type of malignancy causing the biliary obstruction, stage of the malignancy, and presence of metastasis could have all influenced the results. Location of the stricture might also influence the outcomes. Retrospective cohort studies were included in this meta-analysis along with RCTs.

The strengths of this meta-analysis include the high-quality methodology of statistical analysis, high-quality methodology used in individual studies, large number of studies compared to prior meta-analyses, and the total number of patients included in this analysis (*N* = 3532). Newer trials were included in this analysis adding to the data that is already present.

Based on the results from our meta-analysis, preoperative biliary stenting has overall less complications compared to the direct surgery group, especially with the use of internal PBD. This may further translate into less cost burden and suffering for patients. We could speculate that PBD could be indicated in certain clinical scenarios if done under good expertise. PBD could be used in patients with unresectable malignancies, in patients with high surgical risk and unfit for surgery, patients with delay in surgery, and patients awaiting surgery and undergoing neo-adjuvant therapy. Current meta-analysis reached slightly different results compared to previous meta-analysis. This may be due to the results from the newly published studies included in this meta-analysis, which were not included in the prior meta-analysis. The advancement in technological aspects and operative skills of biliary drainage procedures could have also contributed to these results.

There was no mortality difference in both groups; this could probably be due to the underlying disease nature itself. Due to the limited data available on cost-benefit analysis and quality of life, we could not analyze further in this regard.

Studies with statistically significant positive results tend to be published and cited. Additionally, smaller studies may show larger treatment effects compared to larger studies. This publication and selection bias may affect the summary estimates. The bias can be estimated using Egger bias indicators and the construction of funnel plots, whose shape can be affected by bias. In the present meta-analysis and systematic review, bias calculations both Egger [52] and Begg-Mazumdar [53] bias indicators showed no statistically significant bias. Furthermore, analysis using funnel plots were used to represent publication bias among the studies included in the present analysis.

## Conclusions

In patients with malignant biliary jaundice requiring surgery, our analysis showed that the preoperative biliary drainage group had significantly less major adverse effects than direct surgery group. Length of hospital stay was comparable in both the groups. There was no clear mortality benefit with preoperative biliary drainage compared to direct surgery. Subgroup of patients that underwent internal PBD had statistically significant reduction in major adverse effects compared to DS group.
